# MEMOSys: Bioinformatics platform for genome-scale metabolic models

**DOI:** 10.1186/1752-0509-5-20

**Published:** 2011-01-31

**Authors:** Stephan Pabinger, Robert Rader, Rasmus Agren, Jens Nielsen, Zlatko Trajanoski

**Affiliations:** 1 Institute for Genomics and Bioinformatics, Graz University of Technology, Petersgasse 14, 8010 Graz, Austria; 2 Christian Doppler Laboratory for Genomics and Bioinformatics, Petersgasse 14, 8010 Graz, Austria; 3 Division for Bioinformatics, Innsbruck Medical University, Schöpfstrasse 45, 6020 Innsbruck, Austria; 4 Department of Chemical and Biological Engineering, Chalmers University of Technology, SE-412 96 Gothenburg, Sweden

## Abstract

**Background:**

Recent advances in genomic sequencing have enabled the use of genome sequencing in standard biological and biotechnological research projects. The challenge is how to integrate the large amount of data in order to gain novel biological insights. One way to leverage sequence data is to use genome-scale metabolic models. We have therefore designed and implemented a bioinformatics platform which supports the development of such metabolic models.

**Results:**

MEMOSys (MEtabolic MOdel research and development System) is a versatile platform for the management, storage, and development of genome-scale metabolic models. It supports the development of new models by providing a built-in version control system which offers access to the complete developmental history. Moreover, the integrated web board, the authorization system, and the definition of user roles allow collaborations across departments and institutions. Research on existing models is facilitated by a search system, references to external databases, and a feature-rich comparison mechanism. MEMOSys provides customizable data exchange mechanisms using the SBML format to enable analysis in external tools. The web application is based on the Java EE framework and offers an intuitive user interface. It currently contains six annotated microbial metabolic models.

**Conclusions:**

We have developed a web-based system designed to provide researchers a novel application facilitating the management and development of metabolic models. The system is freely available at http://www.icbi.at/MEMOSys.

## Background

With the assembly of the first whole genome sequences in the mid-1990s [1], it became possible to identify all gene products involved in biological processes of an organism [2], and soon it became obvious that a holistic approach can provide answers to relevant mechanistic questions, i.e. by simultaneously studying all processes and dynamic interactions at molecular level in order to define entire pathways and predict the behavior of the investigated systems. The molecular activity of cell components is strongly interconnected and needs to be investigated using a system-level approach to explain physiological characteristics and dynamic behavior [3]. Over and above, recent development of next generation sequencing platforms considerably reduces analysis time and financial effort, which allows a far wider use of genome sequencing in biological and biotechnological research. The task at hand is now to integrate the large amount of data about biological systems into models in order to gain novel insights into their interconnected functionality [4].

A well-established approach to analyze high-throughput results of complex cellular systems is genome-scale metabolic modeling, as it provides a new way for investigating molecular processes [5,6]. A metabolic model aims at assessing the physiological states of an organism by describing it as the sum of all chemical reactions of a particular system [7]. Due to the growing number of sequenced organisms, genome-scale metabolic models are compiled for more and more organisms, including over 20 manually curated bacterial species, yeast and several fungi, as well as components of mammalian cells [6,8,9]. Genome-scale metabolic models have already proven to be valuable for strain engineering which aims at improving production yield and stability [10,11]. Their ability to predict the outcome of gene deletions and prognosticate the adaptation of an organism to new nutritional environments makes them a useful instrument to determine the characteristics of alternative flux distributions [12]. Future applications of metabolic models will contribute to the understanding of microbial genomes and may lead to better diagnostic tests and therapies for human diseases [13].

The reconstruction of a new model can be supported by protein homology comparisons with already existing networks. Therefore, several repositories were created which provide access to established metabolic models (e.g.: BioCyc [14], BioModels [15], BIGG [16]). However, while these databases and web applications allow researchers to download and query metabolic models, none of them provides support for the laborious reconstruction process. Assembling the final version of a model is an iterative process involving many manual steps that generate multiple intermediate versions. The possibility to review all changes, extract previous versions, and use an intuitive interface to create new entries would greatly enhance the construction of new models. Hence, an application supporting the development of a model would be of great help to the community.

Basic requirement for such a model development application is the support of data standards. The most prominent standard to describe biological models is the Systems Biology Markup Language (SBML) [17]. It provides a common intermediate format that can be used to define models in regulatory networks, signaling pathways, gene regulation networks, and metabolic pathways. The work of Herrgård *et al. *[9] focused on combining existing yeast models into one consensus reconstruction and published a SBML compliant model where they laid special emphasis on referencing model components to persistent databases or using database-independent annotations, such as SMILES [18] or InChI [19] strings. These annotations allow the unambiguous identification of components and solve the problem of comparing models where an inconsistent naming schema was applied. Therefore, enzymes should be assigned to distinct Enzyme Commission classification numbers (EC numbers) and may be further linked to established and manually curated databases like KEGG [20]. Moreover, metabolites should be annotated with unique identifiers as defined in the ChEBI database [21] and the KEGG Compound Database [22] to enable unambiguous component identification.

The existence of a unified bioinformatics platform which supports researchers in the construction of new genome-scale metabolic models and offers sophisticated query mechanisms accessing the complete developmental history would be of great interest to the metabolic research community. Therefore, we have developed a new application combining all above mentioned functionalities to support research and development of genome-scale metabolic models. The platform provides customizable data exchange mechanisms using the SBML format, an integrated version control system, and it currently holds six microbial genome-scale metabolic models.

## Implementation

The MEMOSys system was implemented in Java, a platform independent and object-oriented programming language [23]. The application is based on Java Enterprise Edition 5 (Java EE) and uses the JBoss Seam framework [24]. It features a three-tier architecture consisting of a presentation-, logic-, and database- layer. A relational database (PostgreSQL [25] or Oracle [26]) is used as the persistence backend which is accessed by the Hibernate [27] persistence framework. The logic layer consists of ordinary Java objects (POJOs) and Enterprise Java Beans (EJB) deployed on a JBoss [28] application server. The presentation layer is based on Java Server Faces (JSF) [29] and the JBoss Richfaces [30] component library.

The schema of the MEMOSys application has been designed using the Unified Modeling Language (UML) [31] (see additional file 1: databaseDiagram.pdf depicting the database model). The use of a UML representation improves maintainability as the application architecture is outright visible and provides an important part of the system documentation. After finishing the definition of a database layout the reverse engineering mechanism of the Seam framework has been used to create a first scaffold of MEMOSys. The resulting application components were used as a starting point to implement the required functionality.

Envers [32] has been used to provide basic functionality for the version control mechanism. The framework allows easy versioning of persistent classes and is well integrated into Hibernate. For each annotated entity, Envers creates an additional table to store its revisions. A unique revision number is used for all entities to allow retrieving a view of the database at a certain revision. The framework provides a rich API which is used by MEMOSys to search for archived data. In addition, Hibernate native SQL queries are used to fetch versioned entities where the API provided by Envers could not be used.

The libSBML library [33] is used to read and create SBML files. Furthermore, it has been modified to include attributes which were not present in exported files under certain conditions (e.g.: always output stoichiometry information). The modifications included customizing the libSBML source code files and adapting several libSBML wrapper classes.

**Figure 1:**
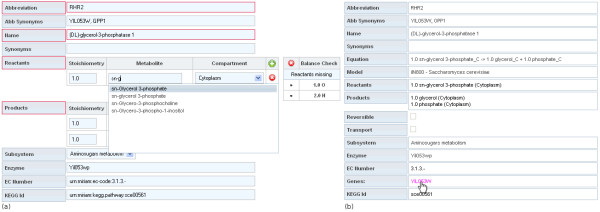
**Edit and view reactions**. The edit view (a) uses a balance check mechanism to check the equilibrium of production and consumption and highlights missing or overproduced components. EC Number and KEGG Id input fields are examples of inserted MIRIAM identifiers. The detail view (b) of a reaction displays the full chemical equation and transforms MIRIAM identifiers into links that open the corresponding external resource page.

The application uses the resource description framework (RDF) and the "Minimum information requested in the annotation of biochemical models" (MIRIAM) [34] notation to annotate components with external references. Each MIRIAM identifier is a single unique string, which unambiguously references an object in an external resource (see Figure 1).

Stored data is secured by a user management system which allows the definition of different user access levels and offers concurrent access in a multi-centric environment [35]. Permission checks are performed at all layers of the application guaranteeing a fine grained and secure authorization mechanism.

## Results

MEMOSys is an application for managing, developing, and storing genome-scale metabolic models of microbial organisms. It is a database-centric Java EE application including a web front-end, a version control system, comparison functionality, and data exchange mechanisms. In the following, the major features of the application are described.

### Data Model

MEMOSys has been designed to map and store all properties of a metabolic model in a database. Reactions are the essential part of a model and their main attributes are name, reactants, and their reversibility. Each reactant is made up of a metabolite, the stoichiometric coefficient for that metabolite, and is assigned to a compartment. Compartments are arranged in a hierarchy which is mapped to the database using references to their parent compartment. Reactions can be assigned to a subsystem which is a representation of a certain metabolic pathway. To support users in the creation or adaptation of reactions, MEMOSys provides a balance check mechanism that validates the elemental composition of consuming and producing reactants (see Figure 1).

In addition to general properties like name and EC number, reactions are linked to citations in order to provide primary literature evidence. Genes and their relationship to other genes and reactions can be described using Boolean operators and hierarchical structures (e.g.: gene1 AND [gene2 OR gene3]). For genes having a reference to the UniProt database [36], the system provides a mechanism to download the amino acid sequence of the transcribed protein and display additional information.

The model is represented in the database by a name, its unique model id, and containing reactions. It specifies an organism and may contain references to an image that graphically represents the metabolic map. Compartments, metabolites, genes, and reactions may include several references to external databases using the MIRIAM notation. MEMOSys automatically transforms MIRIAM annotations to web addresses and displays links to the external references. Moreover, the application includes a mechanism to easily define additional external databases which can be referenced by components. The reference itself can be provided either as a MIRIAM annotation or as plain hyperlink.

### Version control

**Figure 2:**
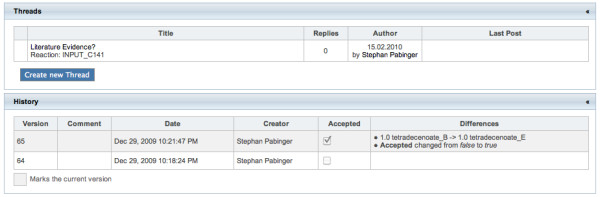
**Entity discussions and history**. The top part of the figure lists the discussions about the current reaction. The lower part shows its history including the latest modifications. For each version a comment, the timestamp, the user, and the differences to the last version are shown. The currently selected version is marked using a gray background color.

**Figure 3:**
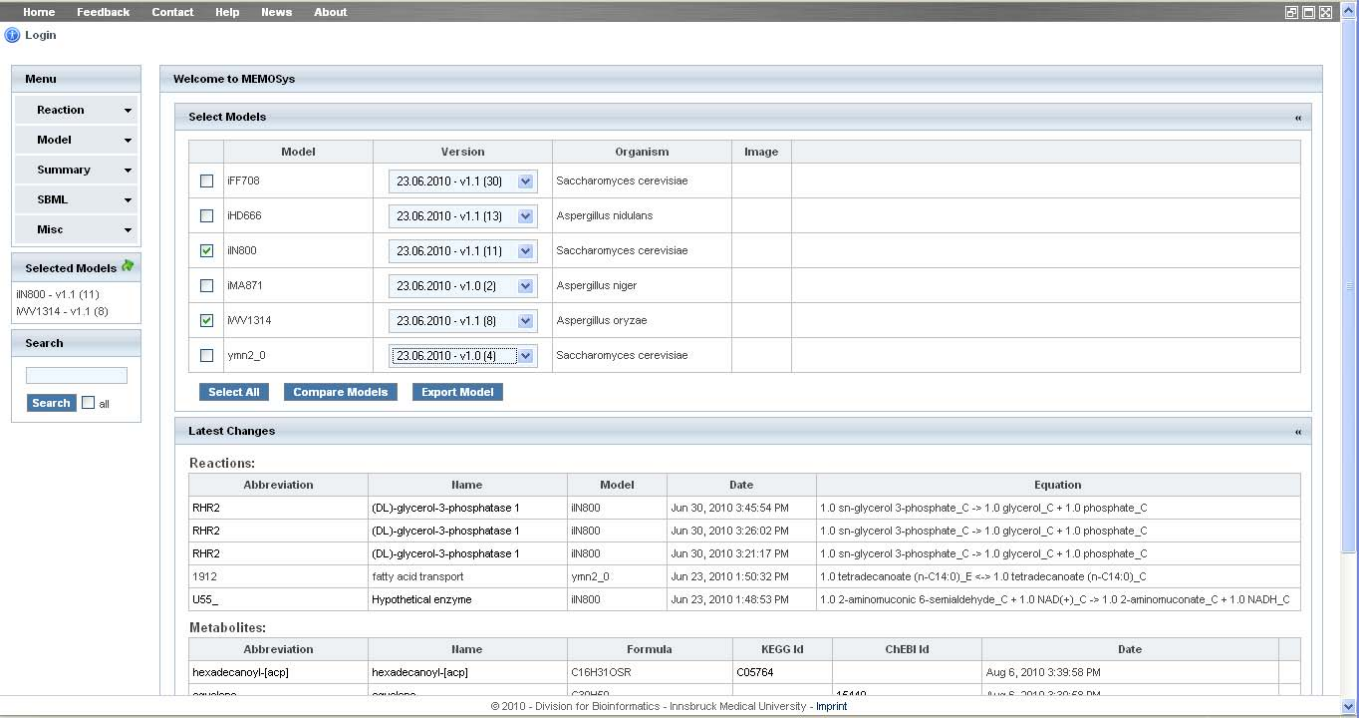
**Home screen**. This figure illustrates the home screen of the application where users can select models and versions used in the reaction view and quick search functionality. The page displays the latest modifications of reactions and metabolites and shows the newest comments on the web board (collapsed in the figure).

The development of a metabolic model is an iterative task producing several intermediate versions until the final model is established. Therefore, the application includes an automatic version control system that stores each modification as a new revision. Except for static information like file uploads, all entities and their references are included in the version control system. This allows researchers to retrieve the complete model at any revision and query, compare, and export previous versions of a model. MEMOSys provides a function to display the history of all version controlled entities including a list of modifications between each version, which allows researchers to track development changes (see Figure 2). The home screen (see Figure 3) of the application lists the latest modifications for metabolites and reactions which gives users a first overview about recent updates.

### Model selection

The home screen of the application displays a list of all available models and allows the selection of specific versions. Since each reaction belongs to a model, the system allows users to restrict reaction queries to currently selected models and examine reactions of only one model or several models at once. The model selection is taken into account by all reaction queries and the quick search functionality.

### Quick search

The quick search field, located beneath the navigation menu on the start page, queries for several entities at once:

• **Reactions **are searched for abbreviation, name, EC number, enzyme, ORF, and KEGG ID.

• **Metabolites **are searched for abbreviation, name, formula, ChEBI ID, and KEGG ID.

• **Genes **are searched for abbreviation, name, EC number, KEGG ID, and UniProt ID

• **Organisms **are searched for name.

For each entity type the result page displays lists of found items and provides links to referenced objects.

### Data exchange

MEMOSys features import and export of metabolic models that are stored in the SBML format.

MEMOSys allows export of all available versions of a model and supports restricting the exported reactions by either including only reactions that are in certain subsystems, or using the result of a reaction query as input for the export mechanism. Furthermore, the export functionality defines three different ways to assign reactions and metabolites to compartments (compartmentalization):

• **Completely Compartmentalized **- reactions and metabolites are assigned to compartments as they are stored in the database.

• **Partially Decompartmentalized **- reactions and metabolites assigned to compartments which are within the cytoplasm (e.g.: peroxisome, nucleus) are reassigned to the cytoplasm. All other compartments are still present in the exported model.

• **Fully Decompartmentalized **- the exported model contains no compartments resulting in an unsegregated system.

Currently, MEMOSys supports export of models into valid SBML files that contain either all stored information about a model or are optimized for usage in the COBRA toolbox [37]. In addition to the SBML export functionality, metabolite and reaction lists can be exported into Excel or PDF files for further use.

### Model comparison

Great attention has been paid to the implementation of a model comparison functionality. The application allows researchers to compare any version of two different models. Moreover, it is possible to compare two versions of the same model to identify development changes. Reactions are compared based on their KEGG ID if available for both reactions. Otherwise the system uses the chemical equation (reversibility, metabolites, and their stoichiometry) for comparison. Metabolites are compared based on their ChEBI ID, KEGG ID, and their name. Genes are compared based on their UniProt ID and their name.

**Figure 4:**
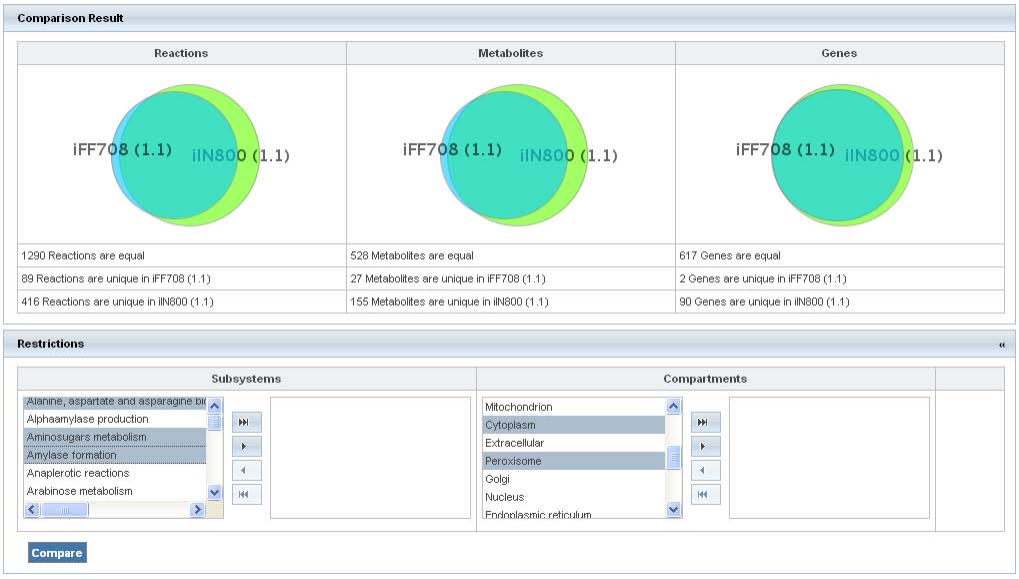
**Visualization of a comparison result**. Displayed is a graphical representation of a model comparison result. It shows the area accurate Venn diagrams for reactions, metabolites, and genes, and provides a short quantitative description of the differences between the models. In the next stage the result list can be restricted by the selection of subsystems or compartments.

The first section of the comparison result displays a summary of the detected differences (see Figure 4) and shows Venn diagrams for reactions, metabolites, and genes. Next, restrictions on the used models can be applied to limit the results to selected compartments and subsystems. In addition to the graphical representation, the application shows detailed lists of equal and unique entities for each model and provides tabs to switch between reactions, metabolites, and genes. Furthermore, reactions, metabolites, and genes are linked to the corresponding pages which display detailed information about the components.

### User access

MEMOSys is designed as a multi-user application which is capable of handling concurrent accesses due to the used enterprise application framework. It defines four different user classes to control data access (see Table 1). Unregistered visitors are allowed to view only accepted, publicly available versions of a model. Registered users are able to browse in addition to publicly available models, accepted versions of assigned models. Furthermore, for registered users certain user settings are persisted in the database to increase the usability of the application. Editors are able to create, update, and delete entities of assigned models in the database. Moreover, they have access to their unpublished models, are allowed to upload files to the web server, and import SBML models into the database. Administrators are editors, which have access to all models, are able to accept modifications of entities, and change the public availability of models.

**Table 1:** User classes in MEMOSys

User class	Browse models	Edit model	Export model	Import model	Modification approval
Unregistered visitors	All accepted versions of publicly available models	-	✓	-	-

Registered users	All accepted versions of publicly available and assigned models	-	✓	-	-

Editor	All accepted versions of publicly available models and all versions of assigned models	All assigned models	✓	✓	-

Administrator	All models	All models	✓	✓	✓

### Supervision

Each modification of an object is at first marked as pending and needs to be confirmed by an administrator. The system provides a clean user interface to accept pending changes. Every time an administrator approves modifications, a new version number is assigned to the model. In addition to the internal revision number provided by the auditing system, models contain a user defined version number which can be set by administrators. MEMOSys differentiates between two access types for models: (a) public available models are visible to all visitors and contain only accepted modifications; (b) assigned models are only visible to registered users and editors of the particular models.

### Web board

The integrated web board allows researchers to create general threads and attach discussions to stored entities. On every entity page a list of currently attached threads is shown, and new discussions can be added to the object. The latest comments of all discussions are displayed on the home screen to quickly update users about new topics. Administrators are able to mark threads as sticky to permanently display them on top of the thread list.

### Model integration

The developed application has been filled with several well-annotated reconstructions of metabolic models. Each reconstruction was manually reviewed in accordance with the model developers and has been improved to be stored as a valid SBML file.

The following models are currently stored in the system:

• **iWV1314**- *Aspergillus oryzae *[38]

• **iMA871 **- *Aspergillus niger *[39]

• **iHD666 **- *Aspergillus nidulans *[40]

• **iFF708 **- *Saccharomyces cerevisiae *[41]

• **iIN800 **- *Saccharomyces cerevisiae *[42]

• **ymn2_0 **- *Saccharomyces cerevisiae *[9]

Additional models will be included in the future. Moreover, authors are encouraged to provide their models in order to enlarge the repository.

## Discussion

We have developed a bioinformatics platform for the management, development, and storage of metabolic models. MEMOSys is aimed at the metabolic research community to facilitate the study of existing metabolic models and ease the collaborative development of new ones.

Blazeck and Alper [43] and Risso et al. [44] state that the development of new metabolic networks is an iterative process where the possibility to reproduce each development step is an important prerequisite. Therefore, MEMOSys features a built-in version control mechanism that automatically stores the complete history of a model. The system allows querying and displaying previous versions of a model and provides the possibility to display the complete history of each component. Since certain use cases demand access to specific versions of a model, the MEMOSys export mechanism is fully integrated into the version control system and provides researchers access to the complete history of a model.

As more and more metabolic models are being generated, the future development of genome-scale models will strongly rely on already existing reconstructions of related organisms. Hence, a flexible and intuitive mechanism to assess the similarity between models is of great importance. For this reason, MEMOSys offers a comparison tool to get an overview of the differences between two models featuring Venn diagrams and allowing restricting the comparison to a selection of subsystems or compartments. As the unique identification of components in metabolic models is a prerequisite for model comparability and scientific collaborations [45], special emphasis has been laid on annotating components with external references using the MIRIAM notation.

Over the past years, numerous methods and toolboxes have been developed to analyze large-scale metabolic networks [37,46-48]. In order to make use of these tools, the application provides sophisticated data exchange mechanisms that allow the export of models into valid SBML files, which can be, for instance, directly imported into the widely-used COBRA toolbox. Furthermore, the application is easily extensible to include file formats of new analysis tools. MEMOSys supports several different ways of compartmentalization which allows researchers to directly use exported models in analysis tools that do not support a fine-grained assignment of reactions to compartments.

The development of new metabolic models is facilitated by using previously inserted, well annotated components that were created during prior reconstruction processes. Furthermore, due to the flexible export mechanism certain parts of existing models can be used as a scaffold for new models. As the development of a new model is often a collaborative task between several different institutes, specific prerequisites are required by the used software. MEMOSys meets these demands by providing many useful development and collaboration features. The application supports the definition of user roles to guarantee that unpublished data is only visible within a specific group. Furthermore, the application offers a feature rich editing system and includes a supervision mechanism which enforces that modifications are approved by a key researcher to maintain a high model quality. The integrated web board facilitates the collaborative development as researchers can create either global threads to discuss general topics, or attach threads to individual objects to debate specific issues of a model or its components. Moreover, MEMOSys supports concurrent access by multiple users and offers an adjustable user management system for data access control.

In contrast to the Pathway Tools [49] software system, which has also been used for the reconstruction of metabolic pathways [50], MEMOSys offers a rich web-based editing functionality for all components and covers change management for relevant model elements by a built-in version control system. Moreover, its specific focus lies on supporting the research and development of metabolic models opposed to the breadth of functionality provided by Pathway Tools. Therefore, MEMOSys is able to offer interfaces and functionalities specifically optimized for the work with genome-scale metabolic models.

The developed bioinformatics platform for genome-scale metabolic models offers a sophisticated version control system, includes a flexible user management system, and is free of charge. Currently existing systems like the BiGG Database or the BioModels database do not actively support researchers during the reconstruction of novel metabolic models by providing an automatic auditing system and a customizable authentication and authorization system. Furthermore, the developed application features a state-of-the-art web front-end and versatile search functionalities, which allow researchers to perform complex search queries on the stored models. MEMOSys is not restricted to a particular organism and is therefore able to manage any metabolic model. It currently contains six well annotated models which have been manually curated to be compliant with the SBML specification.

## Conclusions

In conclusion, the implemented application MEMOSys provides researchers a tool to store, manage, and develop metabolic models. The web interface and the included version control system greatly facilitate the development of new metabolic models and support the study of existing one. Furthermore, the authorization system and the clear definition of user roles allow collaborations across departments and institutions. Due to the flexible and modular software architecture additional tools and new methods can be easily integrated into the application.

## Availability and requirements

• Project name: MEMOSys

• Project home page: http://www.icbi.at/MEMOSys also available through http://sysbio.se/BioMet

• Operating system: Solaris, Linux, Windows, Mac OS X

• Programming language: Java

• Other requirements: Java JDK 1.6.x, Oracle™ 9i or PostgreSQL™ 8.0.x, a server with at least 1 GB of main memory available to the application

• License: GNU AFFERO GENERAL PUBLIC LICENSE

• Any restrictions to use by non-academics: none

The application can be used either by requesting for an account on the existing server or by performing a local installation.

The setup of the application should be completed within a few hours provided the necessary database access rights are granted. We recommend installing the application on a central server by a system administrator. Step-by-step instructions are provided at the project's web site together with the necessary files.

## Authors' contributions

SP designed the application and drafted the manuscript. He was responsible for the design of the database schema, the development of the logic layer, and the implementation of data presentation. RR contributed to conception, design, and implementation of the application and helped drafting the manuscript. RA was responsible for converting models into a valid SBML format and helped designing the application. JN and ZT were responsible for the overall project coordination. All authors gave final approval of the version to be published.

## Supplementary Material

Additional file 1 **MEMOSys database diagram**. Depicted is the UML database diagram of MEMOSys. Click here for file
