# Supplementary figures and images for: MEMOSys: Bioinformatics platform for genome-scale metabolic models
Source: BMC Syst Biol. 2011 Jan 31;5:20. doi: 10.1186/1752-0509-5-20 (PMC3045322; doi:10.1186/1752-0509-5-20)

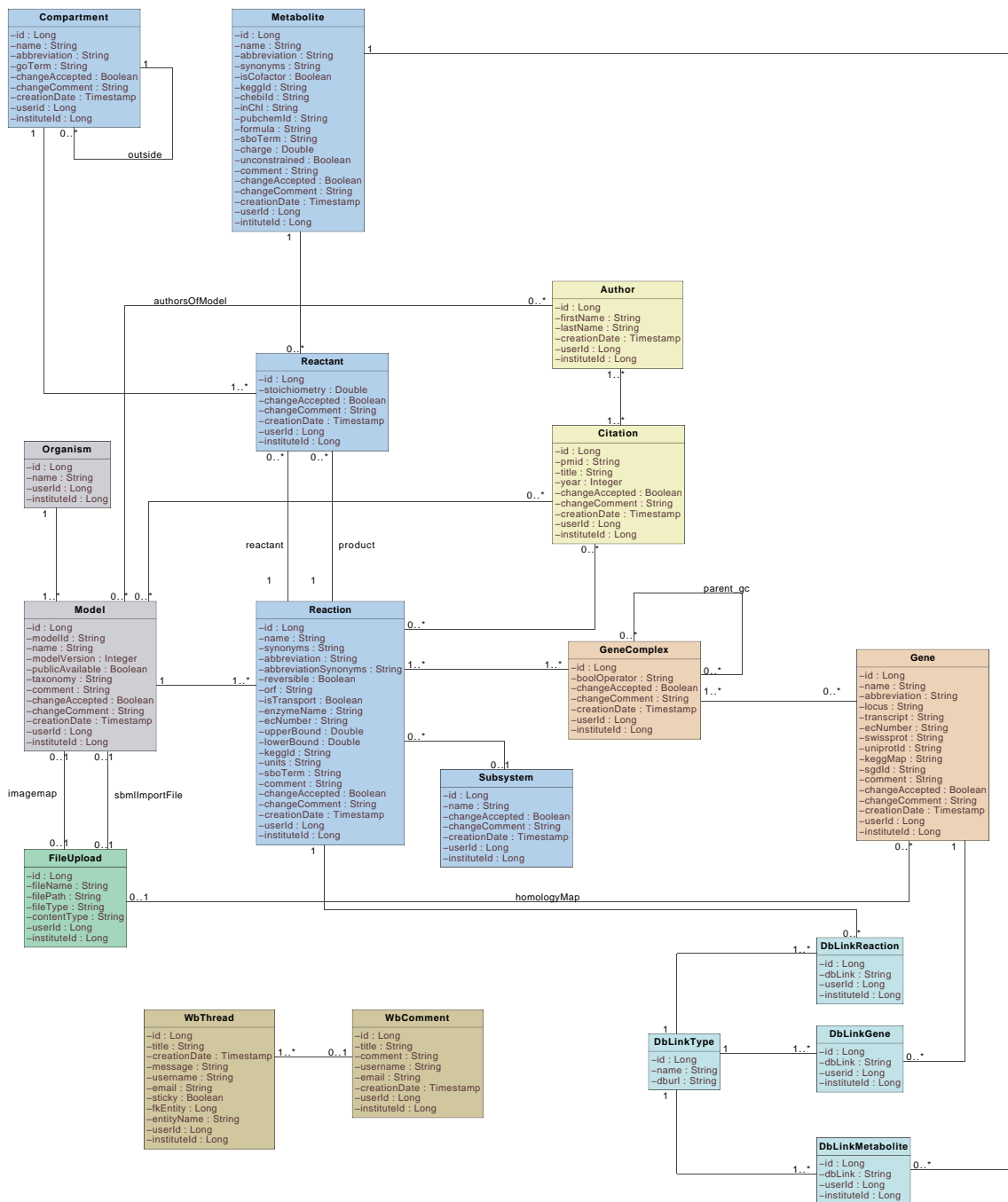

Supplement: Additional file 1 — MEMOSys database diagram. Depicted is the UML database diagram of MEMOSys. [file 1752-0509-5-20-S1.PDF]
